# Gut microbiota from infant with cow’s milk allergy promotes clinical and immune features of atopy in a murine model

**DOI:** 10.1111/all.13787

**Published:** 2019-04-30

**Authors:** Aurélie Mauras, Harm Wopereis, Intan Yeop, Nathalie Esber, Johanne Delannoy, Chantal Labellie, Julie Reygner, Nathalie Kapel, Rob Slump, Tiemen van Eijndthoven, Lieke Rutten, Jan Knol, Johan Garssen, Lucien F. Harthoorn, Marie‐José Butel, Mona Bajaj‐Elliott, Anita Hartog, Anne‐Judith Waligora‐Dupriet

**Affiliations:** ^1^ EA 4065 Faculté de Pharmacie de Paris Université Paris Descartes Paris France; ^2^ UMR‐S 1139, Faculté de Pharmacie de Paris, National Institute for Health and Medical Research Université Paris Descartes Paris France; ^3^ Danone Nutricia Research Utrecht The Netherlands; ^4^ Laboratory of Microbiology Wageningen University Wageningen The Netherlands; ^5^ Great Ormond Street Institute of Child Health University College of London London UK; ^6^ Laboratoire de Coprologie Fonctionnelle, Hôpital Pitié‐Salpêtrière Assistance‐Publique Hôpitaux de Paris Paris France; ^7^ Department of Pharmacology, Faculty of Science, Utrecht Institute for Pharmaceutical Sciences Utrecht University Utrecht The Netherlands; ^8^ Nutricia Research BV Nutricia Advanced Medical Nutrition Utrecht The Netherlands

To the Editor,

Numerous clinical and epidemiological studies suggest an association between abnormal development of the gut microbiota in early life and development of clinical manifestations related to allergy including cow's milk allergy (CMA). Despite advances, at present there is no consensus on a clear signature of a CMA microbiota due to variations in allergic phenotypes and methods used. Moreover, it remains unclear whether the observed microbial alterations are a cause or a consequence of allergy. Here, we report the effects of fecal microbiota transfer (FMT) of a healthy control (HC) and CMA infant in a gnotobiotic murine model of CMA. Detailed methods are provided in Appendix S1.

Infants with CMA (n = 5), under the care of Great Ormond Street Hospital, London, UK, were recruited alongside healthy controls (n = 6) from the community (Tables S1 and S2) (REC 14/LO/0364). Stool samples from the infants with CMA analyzed by 16S rRNA‐gene sequencing showed significant increased bacterial diversity, decreased abundances of *Bifidobacterium* spp., and increased abundances of *Lachnospiraceae* spp., and one of its genera, that is, *Eisenbergiella* (FDR < 0.05), was compared with HC (Figure 1A‐C). These specific gut microbiota signatures corroborate previous findings comparing CMA infants with breastfed HC.^1^, ^2^ Significant increased levels of bacterial‐derived short chain fatty acids (butyrate, iso‐valerate, and iso‐butyrate) were observed in infants with CMA compared with HC (Figure S1). No significant differences in stool pH, acetate, D‐lactate, L‐lactate, sIgA, calprotectin, and eosinophil‐derived neurotoxin levels were recorded (Figure S1). All these parameters were supplemented into a principal component analysis (PCA) of microbial compositions that revealed distinct patterns between CMA and HC (Figure 1D).

**Figure 1:**
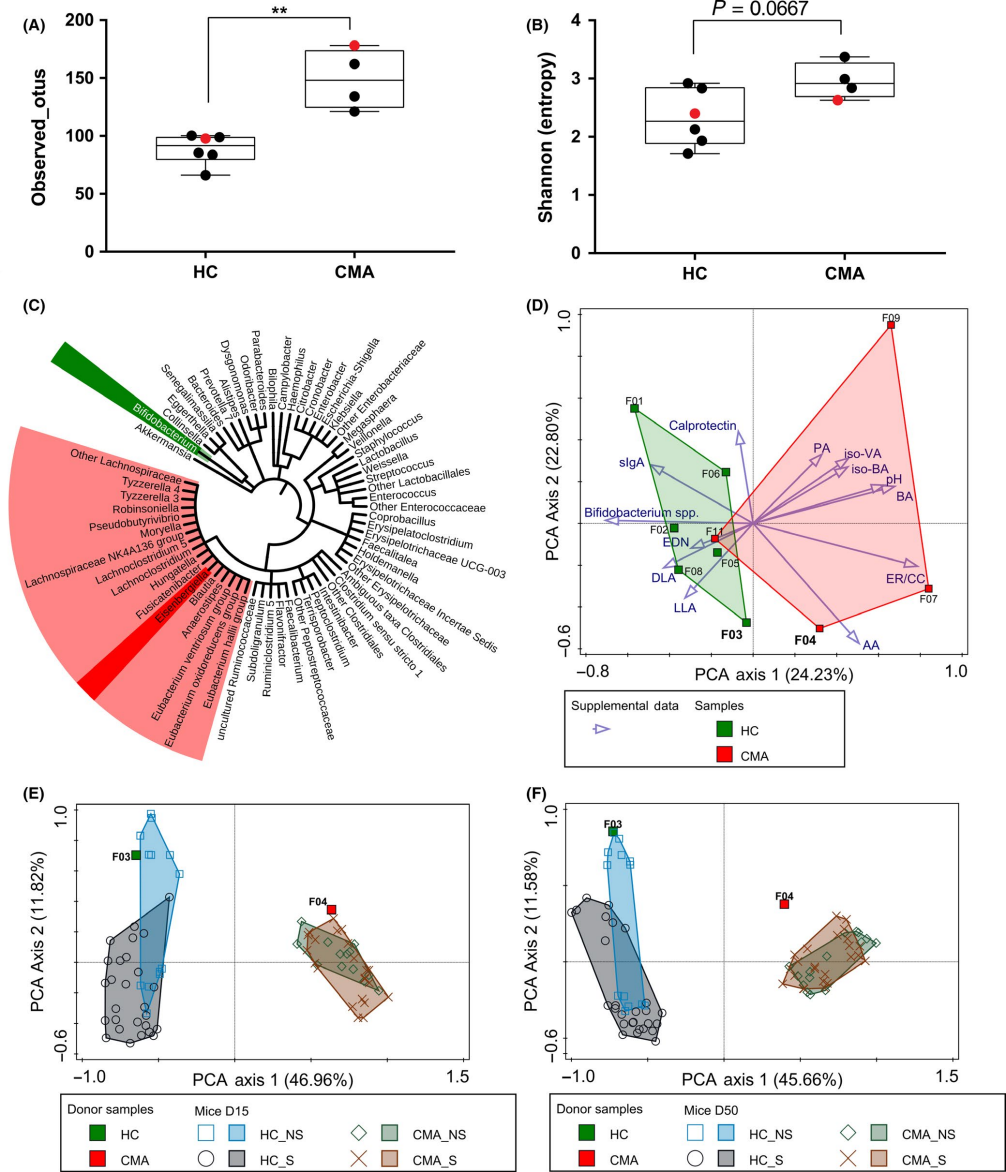
Microbiota of donors and recipient mice. Richness (A) and Shannon diversity (B) of fecal samples from infants with cow's milk allergy (CMA) or without (HC). Red symbols correspond to donors selected for FMT. Cladogram (C) showing discriminant taxa identified at the genus level (dark) and family level (light). PCA (D) of donor microbiota compositions (genus level) supplemented with fecal parameters analyzed. PCA of mice microbiota compositions (genus level) of fecal pellets at D15 (E) and cecal samples at D50 (F). AA, acetic acid; BA, butyric acid; DLA, D‐lactic acid; EDN, Eosinophil‐derived neurotoxin; ER/CC, *Eubacterium rectale*‐*Clostridium coccoides* group; iso‐BA, iso‐butyric acid; iso‐VA, iso‐valeric acid; LLA, L‐lactic acid; PA, propionic acid; VA, valeric acid

We selected 2 infants, 1 with CMA and the other with HC (infants 3 and 4), matched for age (9 and 10 months old), gender (female), and delivery mode (cesarean section) for FMT into three‐week‐old germ‐free mice (C3H/HeN). Following 12 days of microbiota establishment, mice received, once a week for 5 weeks, whole whey protein (WP) and cholera toxin (CT) (sensitized mice; S) or CT only (nonsensitized control; NS) (Figure S2A) (REC CEEA34.AJWD.062.12). Despite an adaptation to the murine host, the main microbial and metabolic signatures of CMA and HC were sustained over the course of the experiment (Figure 1E,F, S2 and S3), validating our model of choice. No differences in microbiota compositions were observed between S and NS mice receiving the same fecal transfer.

Following oral allergen sensitization, CMA microbiota was associated with diarrhea‐related symptoms (Figure 2A), with higher fecal scores (reflecting softer to diarrheic stools/ anal inflammation) that persisted at least 24 hours for the CMA‐S group compared with HC‐S group (*P* < 0.001). No significant differences in fecal scores were recorded between HC‐S and HC‐NS, which may indicate a protective effect of healthy microbiota upon allergen exposure as we and others observed previously.^3^, ^4^ In addition, clinical scores (scratching/ puffiness/ loss of mobility) after oral challenge with β‐lactoglobulin (BLG) were significantly higher in CMA‐S group *versus* CMA‐NS and HC‐S groups (Figure 2B). Minimal differences in mouse mast cell protease 1 (mMCP‐1) and allergen‐specific sensitization markers were observed between the two fecal transfers (Figure 2C‐E), which contrasted with the increases observed in total IgE levels and total IgG1/IgG2a ratio in CMA‐S and CMA‐NS groups compared with HC‐S and HC‐NS groups, respectively (Figure 2F,G). The latter observations were consistent with increased *gata3* mRNA expression in the colon (Figure 2H), which is a marker of Th2 lymphocytes. Interestingly, despite nonsignificant, colonic* fcγRIII* mRNA expression was increased in CMA‐S mice (Figure 2H, *P* = 0.07). FCγRIII is an IgG1 binding/activating receptor, and its increased expression may imply a pathway linked to IgG1 and basophils and the potential development of anaphylaxis.^5^

**Figure 2:**
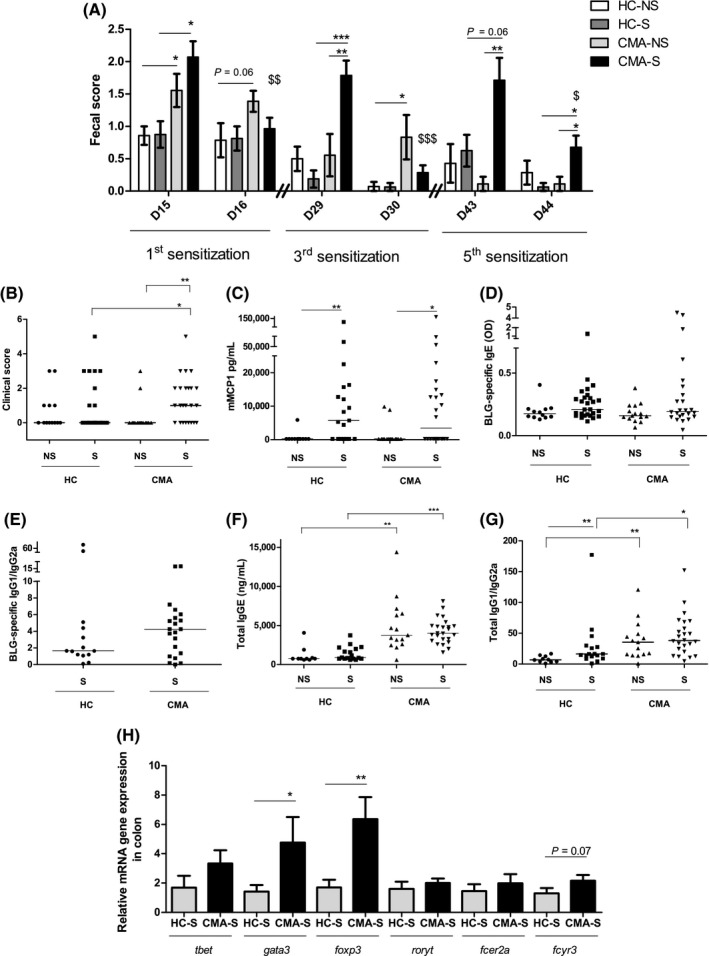
Mice model results (A) Fecal scores: Pellets were collected at D15 & D16, D29 & D30, and D43 & D44. (B) Clinical score, (C) concentration of mMCP‐1, (D) BLG‐specific IgE, (E) BLG‐specific IgG1/IgG2a ratio, (F) total IgE, and (G) total IgG1/IgG2a ratio. (H) Gene mRNA expression in colon of sensitized groups. mRNA expression is relative to HC‐S mice. *P*‐values were calculated using Mann‐Whitney test (*P < 0.05, **P < 0.001, and ***P < 0.0001)

Literature indicates that germ‐free mice have increased total IgE, which can be normalized upon colonization with commensal microbiota until 8 weeks of age.^6^ We found that only colonization with HC‐associated microbiota led to low total IgE levels similar to that of nonsensitized control. This may be due to the observed enrichment of protective bacteria including bifidobacteria and *Anaerostipes* spp. (Figure S2),^7^ the latter of which corroborates the findings by Feehley et al^4^ using a similar mice model. In contrast, CMA microbiota was associated with higher total IgE, a phenomenon that is linked to poor long‐term outcome in atopic dermatitis^8^ or an increased risk of developing other allergic manifestations.^9^

Our findings confirm a Th2‐type immune orientation following FMT with CMA microbiota. This Th2 profile was associated with minimal differences in mMCP‐1 levels or allergen‐specific immunoglobulin levels between the CMA‐S and HC‐S groups, raising the hypothesis that non‐IgE mediated immunity might be at play. Indeed, patients with non‐IgE‐dependent food‐allergy predominantly present gastro‐intestinal tract symptoms. The diarrhea‐like symptoms and signs of colonic inflammation in mice with the CMA‐associated microbiota support this hypothesis.

We noted an increase in colonic *foxp3* mRNA gene expression in CMA‐S group compared with HC‐S group. Foxp3 has been associated with the production of Th2 cytokines in several cell lines, including Foxp3^+^Gata3^+^ cells, as well as with regulatory T‐cells (Treg). If it is associated with Treg cells, the increased *foxp3* expression may reflect a regulatory mechanism in response to the enhanced Th2 profile of the CMA‐S group. Another explanation of the increased *foxp3* expression could be linked to the increase in *Lachnospiraceae* spp. and the associated increase in butyrate (Figure S3), which has been implicated in the induction of Foxp3 Treg cells.^10^ However, in our study, this was not associated with protection against allergic sensitization.

In summary, we demonstrate for the first time that infant microbiota with a low bifidobacteria/*Lachnospiraceae* ratio orients the murine immune system toward a Th2 atopic profile with enhanced clinical symptoms of allergy, showing the pivotal role of the microbiota in allergy. Our study, however, has two limitations: (a) the concomitant medication and elimination diet in infants with CMA, which are the both factors known to influence the gut microbiota, but are also inherent to the medical condition, and (b) the use of only two representative microbiotas to avoid antagonistic effects that might arise by mixing different microbiotas. Despite these limitations, our model system is a valuable tool providing novel insights into the role of gut microbiota in CMA.

## CONFLICTS OF INTEREST

HW, RS, TVE, LR, JK, JG, LFH, and AH are employees of Danone Nutricia Research.
