# Supplementary figures and images for: Gut microbiota from infant with cow’s milk allergy promotes clinical and immune features of atopy in a murine model
Source: Allergy. 2019 Apr 30;74(9):1790–3. doi: 10.1111/all.13787 (PMC6790679; doi:10.1111/all.13787)

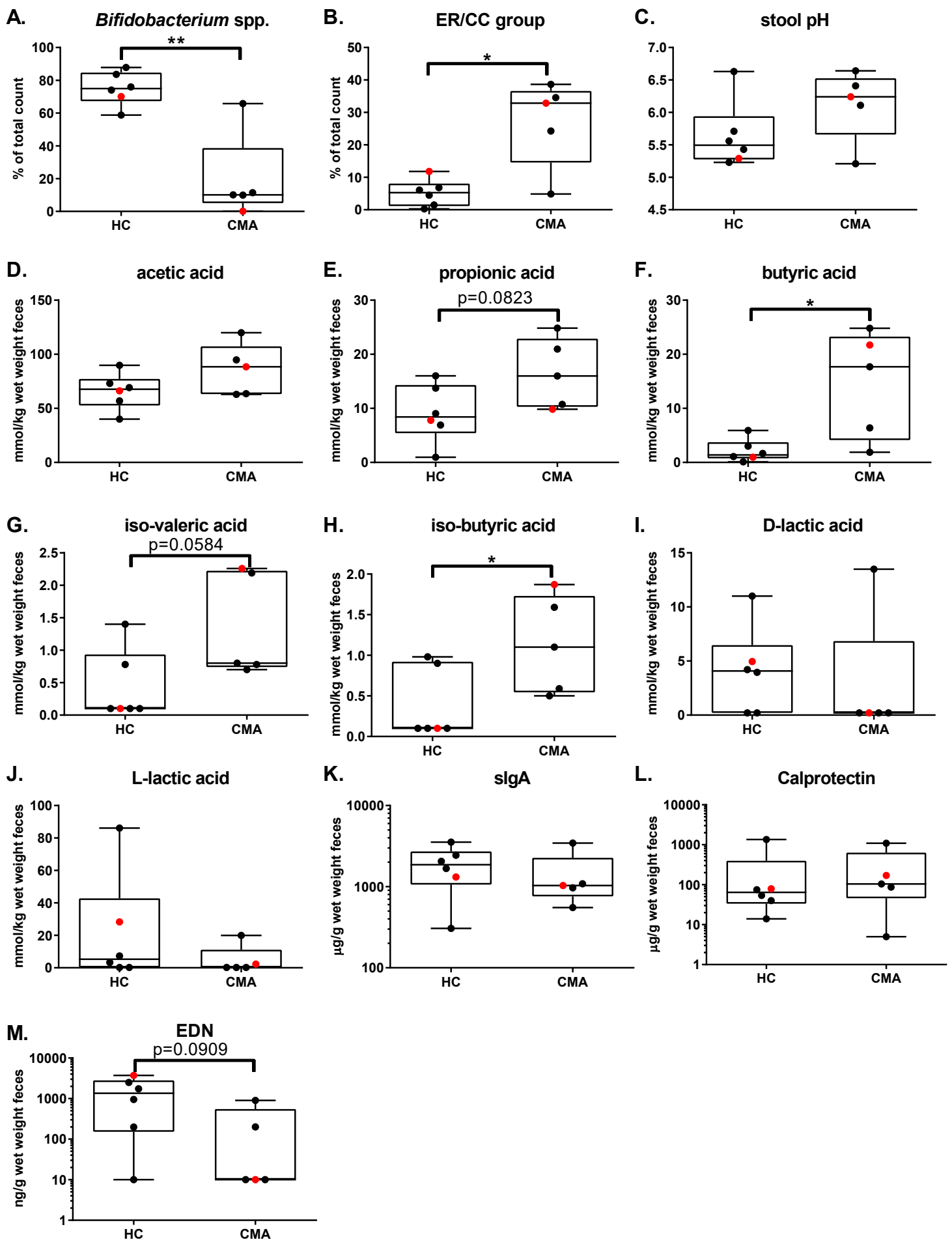

Figure S1

Supplement: Supplementary file 1 [file ALL-74-1790-s001.pdf]

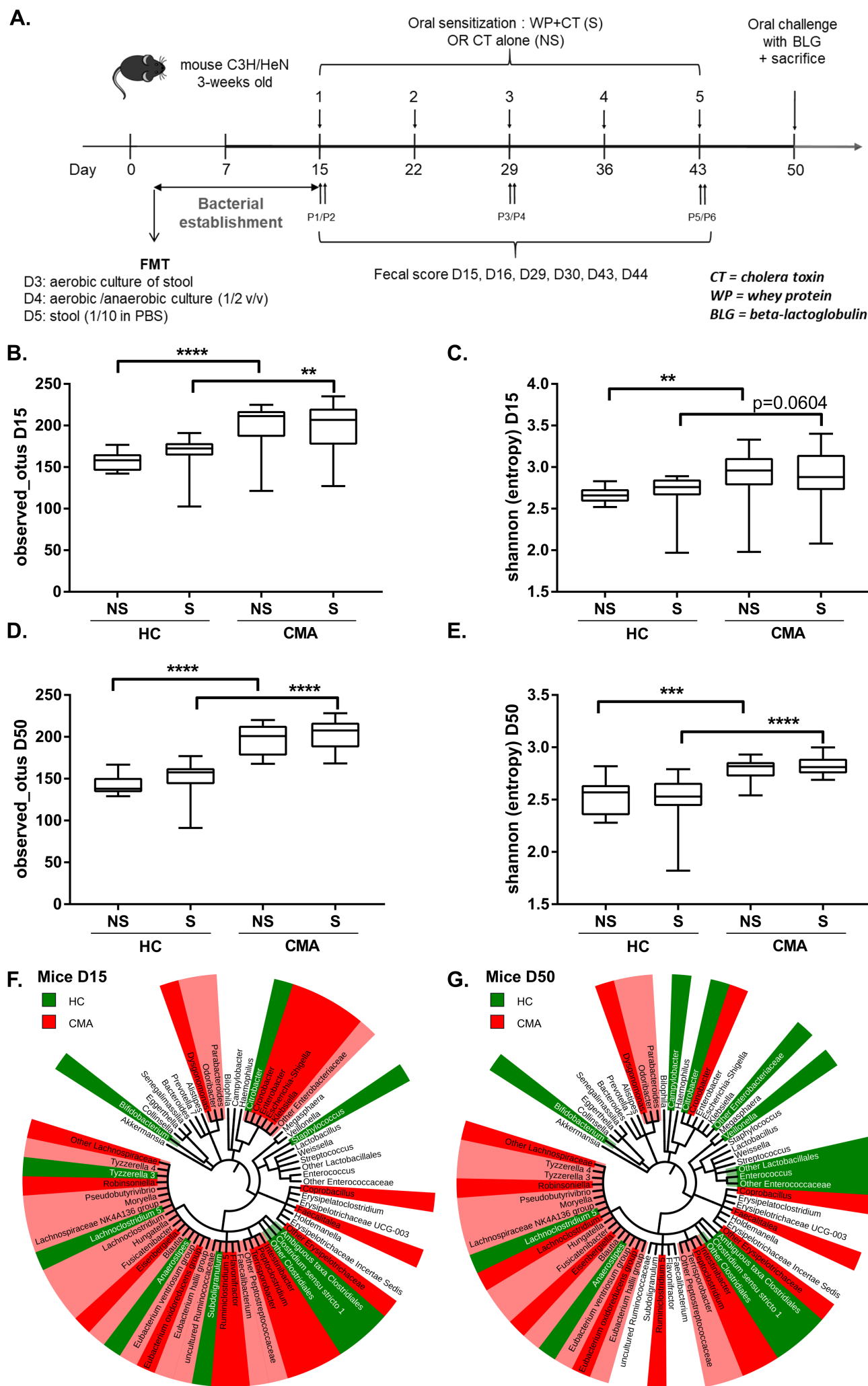

Figure S2

Supplement: Supplementary file 2 [file ALL-74-1790-s002.pdf]

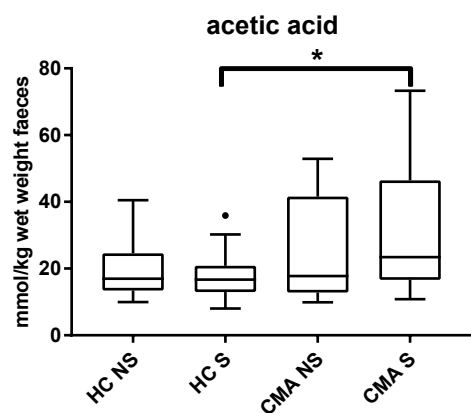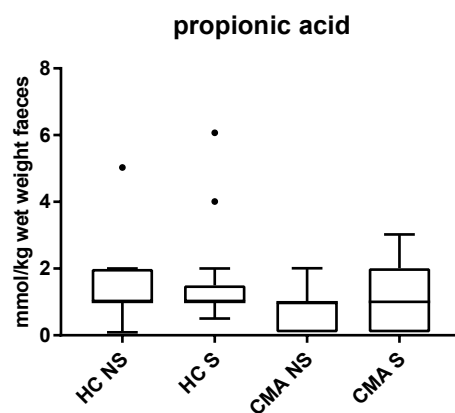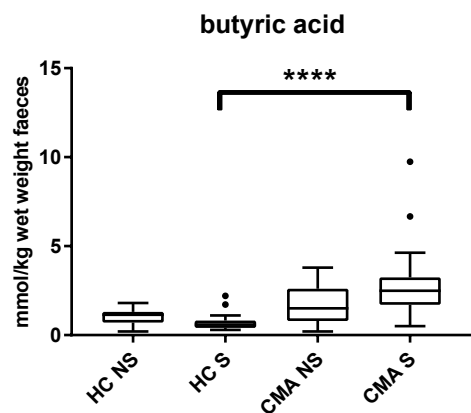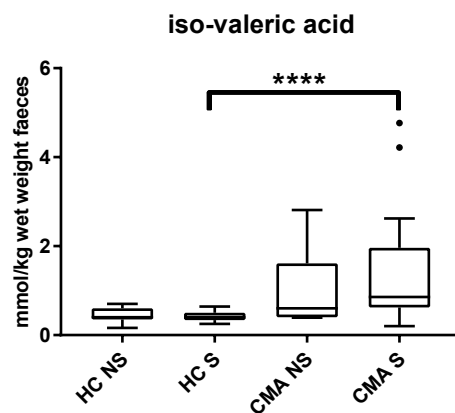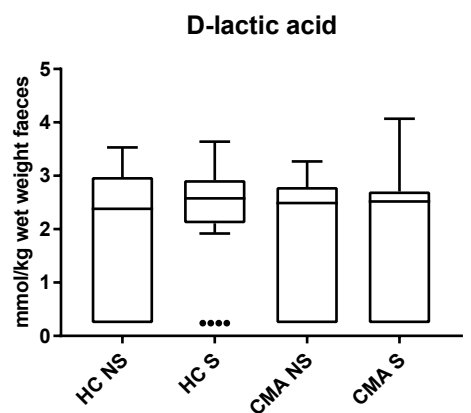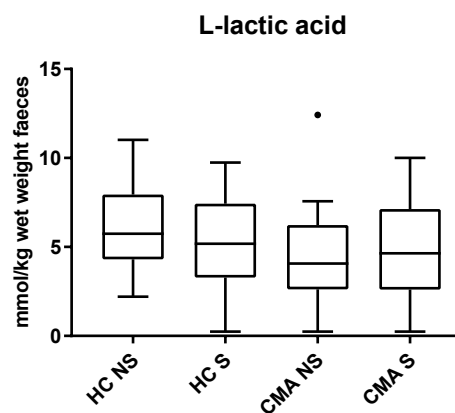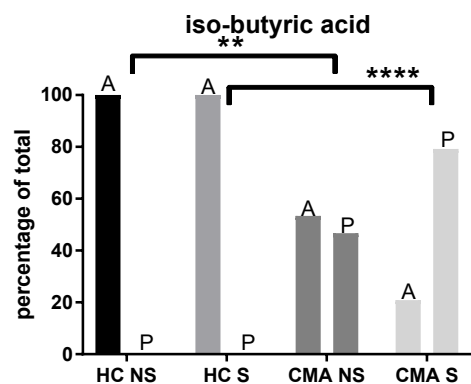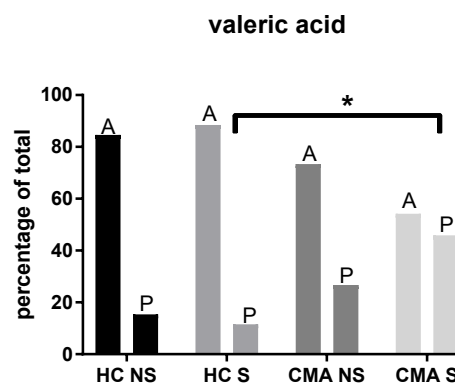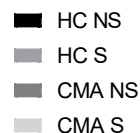

Figure S3

Supplement: Supplementary file 3 [file ALL-74-1790-s003.pdf]
