# Supplementary material for: Gut microbiota from infant with cow’s milk allergy promotes clinical and immune features of atopy in a murine model
Source: Allergy. 2019 Apr 30;74(9):1790–3. doi: 10.1111/all.13787 (PMC6790679; doi:10.1111/all.13787)
Supplement: Supplementary file 4 [file ALL-74-1790-s004.docx]

**Supporting Information**

**Supplementary materials and methods**

**Subjects, allergic workup**

Allergic infants under the care of Great Ormond Street Hospital, London, UK, were recruited alongside healthy infants from the community (REC No 14/LO/0364). The CMA was diagnosed based on clinical presentation (immediate reaction with urticaria) and this was confirmed subsequently following oral challenge. Only term, healthy infants who were fully or partially breastfed, with no family history of atopy, no exposure to antibiotics (in the antenatal or postnatal period), nor any other medications were recruited for comparison with the allergic cohort. Stool from 1 nappy were separated into three containers (1 container with 5 ml 10% glycerol in 0.9% NaCl), then stored and transported at 4^o^C until storage at -80^o^C within 24 hours.

**Colonization of germ-free mice**

The protocol was approved by the Regional Council of Ethics for animal experimentation (Île-de-France, Paris Descartes, CEEA34.AJWD.062.12).

Germ-free C3H/HeN mice (Anaxem, INRA, Jouy-en-Josas, France) were housed in sterile isolators in the animal care facilities of CRP2-UMS 3612 CNRS-US25 INSERM-IRD at the Faculté de Pharmacie de Paris, Université Paris Descartes, France. Mice were allowed *ad libitum* intake of autoclaved water and pellet AIN93G based chow sterilized by γ-irradiation at 50 kGy (ssnifspezialdiäten, Soest, Germany). They received the two selected fecal microbiotas by oral gavage at weaning age (21±3 days of life) producing 2 groups, i.e. healthy infant microbiota-associated mice (HC group) and CMA infant microbiota-associated mice (CMA group). This transfer was performed on 3 consecutive days as described hereafter. Stool was cultured in Tryptone-Glucose-Yeast-Hemin medium at 37°C for 24h under aerobic conditions or 48h under anaerobic conditions (MACS anaerobic cabinet; AES-Chemunex, Bruz, France; N_2_/H_2_/CO_2_; 80:10:10). On day 3 (D3), 100µl of the aerobic culture was administered to mice; on D4, 100µl mix of anaerobic/aerobic culture (2:1, v/v) was administered and on D5, the mice received 100µl of stool diluted in PBS at 1:10 (figure S2A). This sequential inoculation allowed the establishment of extremely oxygen sensitive bacteria (1).

**Oral sensitization and immune challenge**

Each group was divided into 2 subgroups: non-sensitized group included 5 to 6 mice and sensitized groups included 12 to 15 mice. The first subgroup (S group) was sensitized with whey proteins (WP, lacprodan 80, Arla, Lyon, France; 15mg per mouse) and adjuvant cholera toxin (CT) (List Biological Laboratories, Campbell, CA; 10µg mouse^-1^) in PBS. The second subgroup received only CT in PBS as a control (non-sensitized group, NS). Sensitizations were performed by oral gavage once a week for 5 weeks (figure S2A). One week after the last sensitization (D50), all mice received an oral challenge with 60mg of β-lactoglobulin (BLG, Sigma Aldrich, Saint-Quentin-Fallavier, France). Data from 2 independent experiments are shown.

**Evaluation of allergic response**

Clinical scoring was performed on D50, 30 min after the BLG challenge, by two investigators blinded to the sensitization protocol and the mouse groups for 15 min. As previously described (2), allergic symptoms were evaluated based on three criteria: scratching behavior, loss of mobility, and puffiness (including bristled fur, edema around nose and eyes, laborious breathing). Scratching was defined as the number of scratching episodes per 15-min interval as follows: 1-3 episodes=0, 4-5 episodes=1, and >6 episodes =2. Loss of mobility was graded in terms of the duration of absence of any movement as follows: <10 min=0; >10 min = 1, during the 15 min =2. Puffiness was graded as none=0 and puffiness=2. The clinical score was defined as the sum of the three individual scores, and therefore, ranged from 0 to 6.

**Sampling**

Fecal pellets were collected after the 1^st^, 3^rd^ and 5^th^ sensitization for two consecutive days and scored as follows: normal=0, wet=1, glairy and very soft=2, diarrhea=3, no feces or anal inflammation = 1. The fecal score was defined as the sum of the two scores (consistency and inflammation) and therefore, ranged from 0 to 4.

On D50, blood was recovered in K3-EDTA tubes, centrifuged immediately, and plasma was stored at -80°C for immunoglobulin and mast cell protease-1 (mMCP-1) measurements. Spleens and mesenteric lymph nodes (MLN) were used for lymphocyte cultures followed by cytokine dosages. Two-cm of duodenum, jejunum, ileum and colon were collected and stored in RNA-later (Sigma-Aldrich, France) for relative expression gene measurement, and cecum contents stored in dry tube. Samples were stored at -80°C until analysis.

**Gut microbiota and other parameters in infant and mice samples**

The gut microbiota composition and diversity were determined by 16S rRNA-gene sequencing, except for infant nr.10 due to insufficient fecal specimen to perform DNA extraction. DNA was extracted from fecal and cecal samples using a phenol-chloroform based method combined with bead-beating as described previously (3). Infant and mice microbiota compositions were determined by sequencing and bioinformatic analysis as described in detail before (4). Briefly, extracted DNA samples were profiled by sequencing the PCR-amplified V3-V4 regions of the 16S rRNA gene on an Illumina MiSeq instrument (San Diego, USA). Illumina reads were preprocessed, quality filtered, merged and analyzed with an adapted version of the ‘Quantitative Insights Into Microbial Ecology’ (QIIME) v1.9.0 pipeline (5). Sequences were clustered into Operational Taxonomic Units (OTUs) based on 97% sequence identity using VSEARCHv2.4.1 with exclusion of chimeric sequences identified against the RDP gold database (6,7). Taxonomic assignment was performed using the RDP classifier (8) against the SILVA123 database (9). Singleton OTUs, OTUs with eukaryotic assignments, and OTUs with a low relative abundance up to 0.005% were excluded from further downstream analysis. The species diversity (α-diversity) metrics for richness (observed OTUs) and the Shannon index for diversity (10) were calculated using the R-package phyloseq (11) with correction for the differences in sequencing depths by rarefaction.

The 16S rRNA-based sequencing of infant stools was complemented by fecal pH-analysis plus the quantification of *Bifidobacterium* spp. and *Eubacterium rectale*/*Clostridium coccoides* group (ER/CC) by fluorescent *in situ* hybridization (FISH) (4). Additionally, the immune markers eosinophil-derived neurotoxin (EDN) (12), calprotectin (13), and secretory IgA (14) were determined as previously described. Bacterial metabolic activity in both infant and mice samples was assessed through the analysis of short-chain fatty acids (SCFA) (i.e., acetate, propionate, butyrate, iso-butyrate, valerate, and iso-valerate) and lactic acids (D- and L-lactate) as described previously (3).

**Measurement of plasma mouse mast cell protease-1 and sensitization markers**

Plasma mMCP-1, total IgE, IgG1 and IgG2a were measured by ELISA according to manufacturer’s recommendation (Ready-SET-Go!, Ebioscience, San Diego, USA). Measurements of BLG-specific IgE levels were performed by capturing with rat anti-mouse IgE (Pharmingen, BD Biosciences, Le Pont-de-Claix, France) antibody and by detecting with biotinylated BLG (Pierce, Rockford, USA) and streptavidin-horseradish peroxidase (HRP) (Clinisciences, Nanterre, France) as previously described (2). Data were expressed in terms of OD at 450nm. Levels of anti-BLG IgG1 and IgG2a were determined using BLG as the capture antigen, and goat anti-mouse IgG1 and IgG2a-HRP (Southern Biotech, Birmingham, USA) were labeled as detection antibodies as previously described (15).

**Relative expression of genes in the colon**

Total RNA was isolated from 2-cm segment of colon, using an RNeasy Plus universal kit (Qiagen, Courtaboeuf, France). Extracted RNA was treated with DNase I and first-strand cDNA was synthesized using Invitrogen reagents (Thermo Scientific, Illkirch, France) (15).

Quantitative real-time PCR (qRT-PCR) was performed on an ABI Prism 7900HT sequence detection system (Applied Biosystems, Thermo Scientific, Illkirch, France). QuantiTect SYBR green and QuantiTect primer assays (Qiagen) were used to quantify *fcγR3* and *fcer2a* expression. TaqMan gene expression assays with TaqMan universal master mix II (Applied Biosystems) were used to quantify *foxp3*, t-*bet*, *gata3* and *rorγT*. Measurement were performed in duplicate, and gene expression levels were calculated using the 2^-ΔΔ^*^CT^* method (16), where *CT* is the threshold cycle, with the *tata* box (TaqMan) assay as reference gene.

**Statistical analysis**

For statistical analysis and visualization of microbiota community data, Graphpad Prism software version 7.00 for Windows (La Jolla California USA) was adopted for comparisons of species diversity and Canoco 5 software was used for Principal Component Analysis (PCA) (17) with Aitchison log-ratio transformation of bacterial genus compositional data (18). Differential abundance analysis was performed at the bacterial family and genus level using the R-package ANCOM (19) and the iTOL version 4 for visualization of the discriminant taxa in cladograms (20). Corrections for multiple comparisons were controlled using the Benjamini-Hochberg false discovery rate (FDR) method with significance below 0.05 (21). Other fecal and ceacal parameters, mice clinical response data and immune data were analyzed with Graphpad Prism software applying Mann-Whitney test for two-group comparisons and Kruskal-Wallis to compare more groups, with Dunn's correction for multiple testing. A *P* value of less than 0.05 was considered significant.

1. Miquel S, Martín R, Bridonneau C, Robert V, Sokol H, Bermúdez-Humarán LG et al. Ecology and metabolism of the beneficial intestinal commensal bacterium Faecalibacterium prausnitzii. *Gut Microbes* 2014;**5**:146–151.

2. Rodriguez B, Prioult G, Bibiloni R, Nicolis I, Mercenier A, Butel M-J et al. Germ-free status and altered caecal subdominant microbiota are associated with a high susceptibility to cow’s milk allergy in mice. *FEMS Microbiol Ecol* 2011;**76**:133–144.

3. Wopereis H, Sim K, Shaw A, Warner JO, Knol J, Kroll JS. Intestinal microbiota in infants at high risk for allergy: Effects of prebiotics and role in eczema development. *J Allergy Clin Immunol* 2018;**141**:1334-1342.e5.

4. Candy DCA, Van Ampting MTJ, Oude Nijhuis MM, Wopereis H, Butt AM, Peroni DG et al. A synbiotic-containing amino-acid-based formula improves gut microbiota in non-IgE-mediated allergic infants. *Pediatr Res* 2018;**83**:677–686.

5. Caporaso JG, Kuczynski J, Stombaugh J, Bittinger K, Bushman FD, Costello EK et al. QIIME allows analysis of high-throughput community sequencing data. *Nat Methods* 2010;**7**:335–336.

6. Rognes T, Flouri T, Nichols B, Quince C, Mahé F. VSEARCH: a versatile open source tool for metagenomics. *PeerJ* 2016;**4**:e2584.

7. Haas BJ, Gevers D, Earl AM, Feldgarden M, Ward DV, Giannoukos G et al. Chimeric 16S rRNA sequence formation and detection in Sanger and 454-pyrosequenced PCR amplicons. *Genome Res* 2011;**21**:494–504.

8. Wang Q, Garrity GM, Tiedje JM, Cole JR. Naive Bayesian classifier for rapid assignment of rRNA sequences into the new bacterial taxonomy. *Appl Environ Microbiol* 2007;**73**:5261–5267.

9. Pruesse E, Quast C, Knittel K, Fuchs BM, Ludwig W, Peplies J et al. SILVA: a comprehensive online resource for quality checked and aligned ribosomal RNA sequence data compatible with ARB. *Nucleic Acids Res* 2007;**35**:7188–7196.

10. Shannon CE. The mathematical theory of communication. 1963. *MD Comput* 1997;**14**:306–317.

11. McMurdie PJ, Holmes S. phyloseq: an R package for reproducible interactive analysis and graphics of microbiome census data. *PLoS ONE* 2013;**8**:e61217.

12. Kalach N, Kapel N, Waligora-Dupriet A-J, Castelain M-C, Cousin MO, Sauvage C et al. Intestinal permeability and fecal eosinophil-derived neurotoxin are the best diagnosis tools for digestive non-IgE-mediated cow’s milk allergy in toddlers. *Clin Chem Lab Med* 2013;**51**:351–361.

13. Huet F, Abrahamse-Berkeveld M, Tims S, Simeoni U, Beley G, Savagner C et al. Partly Fermented Infant Formulae With Specific Oligosaccharides Support Adequate Infant Growth and Are Well-Tolerated. *J Pediatr Gastroenterol Nutr* 2016;**63**:e43-53.

14. Scholtens PAMJ, Alliet P, Raes M, Alles MS, Kroes H, Boehm G et al. Fecal secretory immunoglobulin A is increased in healthy infants who receive a formula with short-chain galacto-oligosaccharides and long-chain fructo-oligosaccharides. *J Nutr* 2008;**138**:1141–1147.

15. Neau E, Delannoy J, Marion C, Cottart C-H, Labellie C, Holowacz S et al. Three novel candidate probiotic strains with prophylactic properties in a murine model of cow’s milk allergy. *Applied and Environmental Microbiology* 2016;**82**:1722–1733.

16. Livak KJ, Schmittgen TD. Analysis of Relative Gene Expression Data Using Real-Time Quantitative PCR and the 2−ΔΔCT Method. *Methods* 2001;**25**:402–408.

17. Smilauer P, Lepš J. *Multivariate Analysis of Ecological Data using CANOCO 5*. 2nd ed. Cambridge: Cambridge University Press 2014 http://ebooks.cambridge.org/ref/id/CBO9781139627061 (accessed 19 Aug2018).

18. Aitchison J. Reducing the dimensionality of compositional data sets. *Mathematical Geology* 1984;**16**:617–635.

19. Mandal S, Van Treuren W, White RA, Eggesbø M, Knight R, Peddada SD. Analysis of composition of microbiomes: a novel method for studying microbial composition. *Microb Ecol Health Dis* 2015;**26**:27663.

20. Letunic I, Bork P. Interactive Tree Of Life (iTOL): an online tool for phylogenetic tree display and annotation. *Bioinformatics* 2007;**23**:127–128.

21. Benjamini Y, Hochberg Y. Controlling the False Discovery Rate: A Practical and Powerful Approach to Multiple Testing. *Journal of the Royal Statistical Society Series B (Methodological)* 1995;**57**:289–300.

**Table S1**: Patients characteristics. a M=Male, F=Female; b HC=healthy control, All =allergic; c NVD = normal vaginal delivery, LSCS = lower segment caesarean section, El= Elective; d Previous medicine including antibiotics. In bold character, selected donor infant for fecal microbiota transfer to the murine model.

| N° | Sex^a^ | Age collection  (month) | Status^b^ | Delivery^c^ | Family History atopy | Maternal Antibiotics | Previous medecine^d^ | Current medecines | % Breast feeding | Feed | When started feed | Age weaned |
| --- | --- | --- | --- | --- | --- | --- | --- | --- | --- | --- | --- | --- |
| 1 | M | 7 | HC | NVD | No | No | No | No | 100 | NA | NA | 5.5 |
| 2 | F | 5.5 | HC | NVD | No | No | No | No | mostly (1 bottle/day) | Aptamil | birth | 5 |
| **3** | **F** | **9** | **HC** | **LSCS** | **No** | **No** | **No** | **No** | **2x/day** | **Aptamil** | **1 week** | **5.5** |
| 5 | F | 7 | HC | NVD | No | No | No | No | 100 | Breast Feed | birth | 5 |
| 6 | M | 9 | HC | NVD | No | No | No | No | 100 | NA | NA | 4.5 |
| 8 | M | 9 | HC | El LSCS | No | No | No | No | 100 | Breast Feed | NA | 6 |
| **4** | **F** | **10** | **All** | **LSCS** | **Yes** | **No** | **Ranitidine, Lansoprazole** | **Montelukast, Atrovent, Salbutamol, Abidec, Sytron** | **100 (3 months)** | **Nutramigen AA** | **4.5** | **4.5** |
| 7 | F | 16 | All | El LSCS | Yes | No | Infacol, Colic ease | No | 100 | Neocate | 5 weeks | 6 |
| 9 | F | 16 | All | El LSCS | Yes | No | Paracetamol | No | 100 | Neocate | 5 months | 6 |
| 10 | F | 7 | All | NVD | Yes | No | Paracetamol | No | 100 | Neocate | 6 | 6 |
| 11 | F | 12 | All | NVD | Yes | No | Gaviscon, Ranitidine, Omeprazole | Ketotifen | 100 | Neocate | 4 months | 6 |

**Table S2:** Characteristics of CMA infants. CMPA=cow’s milk protein allergy; GOR= gastroesophageal reflux. The CMA was diagnosed based on clinical presentation (immediate reaction with urticaria) and this was confirmed subsequently following oral challenge.

|  | **Patient 4*** | **Patient 7** | **Patient 9** | **Patient 10** | **Patient 11** |
| --- | --- | --- | --- | --- | --- |
| Age (months) | 10 | 16 | 16 | 7 | 12 |
| Sex | Female | Female | Female | Female | Female |
| Clinical manifestations | Urticarial rash, vomiting/GOR | Immediate vomiting, rash, abdominal pain, blood per rectum | Urticarial rash, vomiting, faltering growth, loose stools (with blood and mucus) | Urticarial rash, vomiting | Immediate swelling and rash, vomiting/GOR, faltering growth |
| Personal atopic past history | CMPA, wheat, egg, soya, reactive airway disease | CMPA, egg, wheat, soya, seafood, beef, eczema | CMPA, egg, wheat, soya, shellfish, nut, eczema, chesty | CMPA | CMPA, egg, wheat, soya, rice, eczema, constipation |
| Familial atopic past history | Yes | Yes | Yes | Yes | Yes |
| Final diagnosis | Multiple Food Protein Allergies | Food protein-induced enteropathy syndrome | Multiple Food Protein Allergies | CMPA | Multiple Food Protein Allergies |
| Elimination Diet | Nutramigen AA® | Neocate® | Neocate® | Neocate® | Neocate® |

*Post-exclusive breast feeding, infant #4 developed urticarial rash and classic CMA symptoms when challenged with dairy on repeat occasions; with symptom resolution on exclusion. Infant 4 was stable (amino acid-based formula with dairy/egg/wheat/soya exclusion) when FMT sample was obtained.

**Figure S1:** Fecal markers analyzed in infant donor samples. Box-whisker plots with FISH quantified levels of *Bifidobacterium* spp. (A), and *Eubacterium rectale* – *Clostridum coccoides* (ER/CC) group (B), and stool pH (C). Concentrations of fecal SCFAs, except for valeric acid due to majority of samples had undetectable levels, and lactic acids are summarized (D-J), as well as the concentration of secretory IgA (sIgA) (K), Calprotectin (L) and eosinophil-derived neurotoxin (EDN). Final selected donors for fecal transfer to mice model are highlighted in red. Statistics comparing healthy control (HC) with cow’s milk allergic (CMA) infants were performed using Mann-Whitney test. *p<0.05, ** p<0.001.

**Figure S2:** Microbiota establishment in murine model of CMA (A.). Richness and Shannon diversity analysed in fecal pellets collected at D15 (B and C) and in cecum content collected at D50 (D and E). Cladogram with discriminant taxa identified in mice at D15 (F) and at D50 (G). Taxa that were differentially abundant when comparing HC-mice with CMA-mice are highlighted at the genus (darkest colour) and family level (lighter colour). Green colours indicate an increase of relative abundances in HC, and red colours indicate increased relative abundances in CMA.

**Figure S3:** Levels of SCFAs and lactic acids in cecal content mice (D50). Box-whisker plots with the concentrations of acetate (A), propionate (B), butyrate (C), iso-valeric acid (D), D-lactic acid (E) and L-lactic acid (F). The detection of iso-butyrate (G) and valerate (H) are presented as percentage of samples with detectable levels (P) and undetectable levels (A). Statistics comparing the HC-groups with the CMA-groups were performed using Kruskal-Wallis test with Dunn’s correction for multiple comparisons and applying fisher’s exact test for presence (P) -absence (A) data. *p<0.05, ** p<0.001 ,***p<0.0001, ****p<0.00001.
